# Magnetic Resonance-Guided Laser Induced Thermal Therapy for Glioblastoma Multiforme: A Review

**DOI:** 10.1155/2014/761312

**Published:** 2014-01-16

**Authors:** Sarah E. Norred, Jacqueline Anne Johnson

**Affiliations:** ^1^Biomedical Engineering Department, University of Tennessee, Knoxville, TN 37996, USA; ^2^University of Tennessee Space Institute, Tullahoma, TN 37388, USA

## Abstract

Magnetic resonance-guided laser induced thermotherapy (MRgLITT) has become an increasingly relevant therapy for tumor ablation due to its minimally invasive approach and broad applicability across many tissue types. The current state of the art applies laser irradiation via cooled optical fiber applicators in order to generate ablative heat and necrosis in tumor tissue. Magnetic resonance temperature imaging (MRTI) is used concurrently with this therapy to plan treatments and visualize tumor necrosis. Though application in neurosurgery remains in its infancy, MRgLITT has been found to be a promising therapy for many types of brain tumors. This review examines the current use of MRgLITT with regard to the special clinical challenge of glioblastoma multiforme and examines the potential applications of next-generation nanotherapy specific to the treatment of glioblastoma.

## 1. Introduction

Glioblastoma multiforme (GBM) is the most common type of malignant primary brain tumor in adults, with over 22,000 estimated diagnoses in 2012 [1]. GBM, often referred to as simply “glioblastoma,” presents a unique clinical challenge in that these tumors generally have strong resistance to traditional therapies, can spread aggressively to other areas of the brain, and may be localized in susceptible areas such that the tumor may not be treated without causing damage to adjacent healthy tissue. In addition, the tumors themselves are characterized by heterogeneous areas of necrotizing tissue and peritumoral edema and often are asymptomatic until reaching a large size. As such, the prognosis is very poor, with a five-year relative survival rate no higher than 17% for patients aged between 20 and 44, and progressively lower for older patients [2].

Surgery is usually indicated as the first stage of treatment, along with chemotherapy and radiation therapies. Though near-complete resection of the tumor usually results in longer survival times, recurrence is very common. Many patients, such as those with deep-seated or recurrent tumors, may not be candidates for surgical debulking. In the last decade, laser induced thermal therapy (LITT) has been used as an alternative treatment for several types of brain tumors, including patients with recurrent glioblastoma who were not candidates for a second resection procedure [3]. LITT as a cancer treatment is a thermocoagulative therapy whereby laser irradiation is introduced into a tumor via percutaneous insertion of an optical fiber. The ablative heat generated causes necrosis in the tumor. This procedure has been used clinically in many tissues, including the brain [3–11], liver [12], lung [13], bone [14], and prostate [15].

In addition to having many flexible applications in other therapies, LITT is useful as a cancer treatment due to its minimally invasive debulking approach, repeatability, and compatibility with magnetic resonance imaging (MRI) and that it can be done concurrently with other treatments [4, 8]. It is furthermore useful specifically as a neurosurgical treatment as it produces lesions of a predictable size, does not use ionizing radiation, and can be performed with the patient awake [4, 8, 9]. This paper intends to review current clinical uses of LITT in the treatment of glioblastoma, as well as to evaluate LITT-based therapies, which may have application in the treatment of glioblastoma in the future.

## 2. Clinical Use of LITT in Gliomas

LITT has been used to treat many types of brain tumors, including metastatic brain tumors [7], astrocytoma, meningioma, ependymoma, chordoma, hemangioblastoma, and glioblastoma multiforme. It is as yet an uncommon therapy, though it is becoming increasingly popular as an alternative treatment to traditional resection and radiation therapies. Research is limited to areas that have available infrastructure for LITT application, though appropriate laser sources, image analysis, and thermal analysis software are each becoming more commercially available. Some of the research on the use of LITT in glioblastoma has been done concurrently with brain tumors of other types; a few studies have focused specifically on recurrent glioblastoma.

### 2.1. Laser Interaction with Brain Tissue

Prediction of thermal influence and lesion size caused by laser-tissue interaction is highly relevant to the treatment of glioblastoma as damage to healthy brain tissue can cause significant neurological deficits in patients. The laser interactions in LITT are predominantly thermal and dependent on the effects of applied laser power. Photons emitted from the optical fiber are absorbed by chromophores in the tumor, causing excitation and the subsequent release of thermal energy [16]. If the heat produced is maintained above a certain threshold temperature at which proteins begin to denature, the tissue irreversibly coagulates and the optical properties (most significantly, the absorbance properties) of the tissue change. It is important to carefully monitor the temperature induced by the applicator device, as too quick or large of an increase in temperature may cause carbonization—this increases the absorption of the tissue substantially, thus limiting the potential light penetration. Overheating may also induce vaporization in the target tissue, resulting in an explosive rupture one article has labeled the “popcorn effect” [17].

The types of lasers commonly used for LITT are either continuous-wave neodymium-doped yttrium aluminum garnet (Nd:YAG) lasers (*λ* = 1064 nm) or diode lasers (*λ* = 800–980 nm) which operate at a wide range of powers [4]. Nd:YAG lasers in particular are indicated for use in soft tissue with high blood perfusion, which demonstrate the highest penetration depths at wavelengths between 1000 and 1100 nm [6]. As brain tissue, especially white matter, has a high rate of perfusion [5], the common and relatively cost-effective Nd:YAG laser is an excellent choice for LITT in brain tumors. These wavelengths also lie within the near infrared (NIR) “therapeutic window,” where scattering is significantly higher than absorption, allowing for optimal penetration into the tissue [16]. Diode lasers at a wavelength of 980 nm have been used in a few studies involving LITT in the brain, specifically chosen due to the local maximum in water absorption at this wavelength [8, 9]. Diode lasers also have relevance to certain nanoparticle therapies excited at these wavelengths.

In order to effectively estimate light penetration and thus lesion size, it is highly important to estimate the optical properties of the brain tissue, most importantly the tissue absorption coefficient. Though values vary for different brain tissues, in all cases, the absorption of coagulated tissue is generally greater than that of noncoagulated tissue [18].

### 2.2. Optical Fiber Applicators

Laser irradiation is introduced into the tumor via an optical fiber, usually of a diameter of about 600 *μ*m, of which ~1 cm of the tip is exposed. This setup generates a uniform ellipsoid lesion along the axis of the fiber [8]. Previously, only this bare tip was used for LITT, resulting in significant overheating effects (carbonization and vaporization) at the tissue surface [11, 16]. In addition, the fiber itself can potentially be damaged in cases of extreme overheating. When this occurs, similar to the effects of carbonization and vaporization, the increase in local absorbance does not allow for good penetration, and coagulation is limited to the effects of thermal conduction.

**Figure 1:**
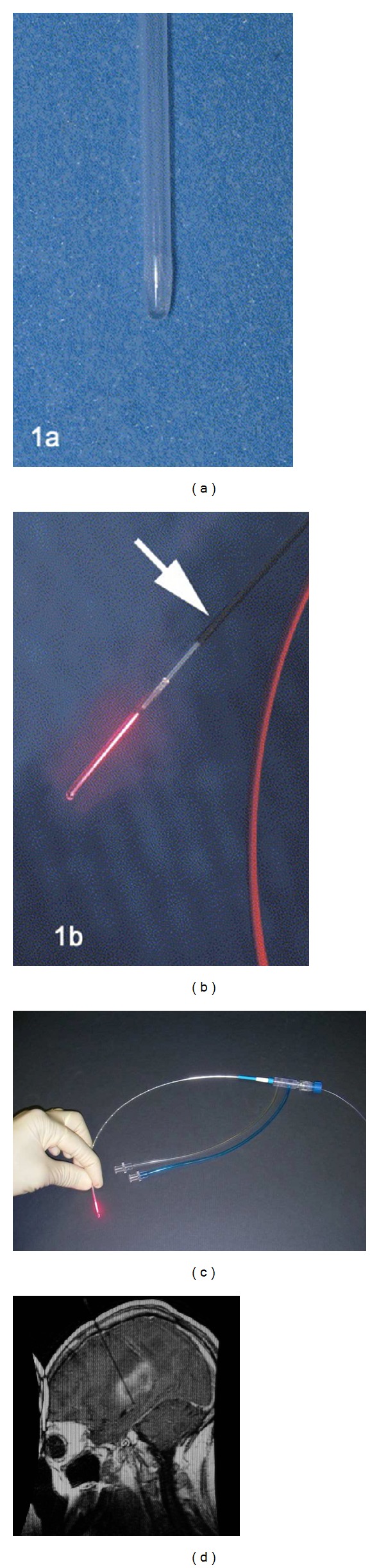
((a), (b)) Visualase applicator tip used in initial clinical investigation of LITT in GBM patients [4]. (a) Fiber protection sheath. (b) Illuminated fiber tip, arrow notes magnetite particle coating used for visual reference during MR imaging [4]. (c) Most recent iteration of Visualase laser applicator, shown with cooling catheter attachment. (d) MR reference image showing the insertion of the entire length of the applicator [9].

Enclosing the fiber in a diffusion sheath allows for the power density to be reduced across the breadth of the tip sheath, allowing for a higher laser power to be used [8]. The sheath surface furthermore provides a nonstick surface such that the fiber may be moved within the coagulation tract easily. More important to recent research and to the use of LITT in glioblastoma is the inclusion of a “cooling catheter,” a sheath-like device which cools the fiber with a constant stream of fluid (trials in the brain have used room-temperature water or saline) [8, 9] which circulates through the lumen of the sheath. The heat transfer induced by the cooling catheter lowers the temperature of both the fiber and the surrounding tissue, allowing the use of much higher power laser systems without inducing carbonization. See Figure 1.

Though only a small portion is used for LITT, the fiber itself is often very long as the laser source must be located outside of the MRI room when MR-guided imaging is used for the fiber placement. Specific paramagnetic markings added on the applicator allow for easy viewing of the location of the fiber tip when imaging the brain [9].

Perhaps most significant to the treatment of GBM is the relative ease at which multiple fibers may be used to attack large and irregularly shaped tumors, which may be used simultaneously or in tandem on a specific area. In principle, the power settings and thermal impact of each fiber can be controlled individually, allowing for highly tuned ablative treatment of tumors with irregular shape [8, 9] and potentially for those with heterogeneous optical properties. A practical concern when using this method in the brain, however, is appropriate spacing of the fiber entry sites to avoid collisions between bone anchors used to hold the fibers in place [9]. In this same case, the fiber tips were kept at least 1 cm apart while in the brain.

However, a complication was noted in one case of multiple fiber usage in a GBM tumor with a very large volume (~70 cm^3^); LITT was attempted with three simultaneous lasers, causing postablation refractory edema that could not be controlled medically and thus required a hemicraniectomy. The investigators assert that control of edema volume should be a priority when large ablation volumes are planned. Though this case had the largest amount of fibers used in a single treatment, the same study had multiple cases where two fibers were used simultaneously without complications [9]. It should be noted, however, that in a similar study using similar thermal therapy and the same MR-guidance system, a patient with recurrent GBM received treatment with 3 fibers on a significantly smaller tumor but did not experience complications [8]. Furthermore, no significant postoperative complications were reported in any patients in a previous study of LITT in recurrent GBM using a Nd:YAG laser along with MR-guidance; this study included a number of patients with large tumor volumes (33.2 cm^3^, 45.5 cm^3^, and 79.8 cm^3^) [4]. Though it may be difficult to draw salient conclusions from individual results across different tests, it may be inferred from the latter examples that patients need not necessarily be excluded from multifiber therapy on the basis of lesion size alone.

#### 2.2.1. Fiber Placement and Thermal Imaging

As correct fiber placement is highly significant to the development of a treatment plan for GBM and the preservation of healthy neural tissue, it is important that all aspects of the therapy are compatible with the imaging technique used in the procedure. Imaging methods for LITT include ultrasound, computed tomography, fluoroscopy, and MRI [16]. In MRgLITT (magnetic resonance-guided laser induced thermal therapy), MRI information is used not only to develop a therapy plan and to accurately place the laser fiber (introduced into the brain using a placement catheter clamped by a plastic skull bolt), but also for the analysis of heating inside the brain tissue using MRTI (magnetic resonance temperature imaging).

An MR-guided technique is well suited for use in brain based tumors as MRI provides excellent definition and contrast in soft tissue, quick, real-time results, and the ability to monitor many different factors, including the local temperature of the coagulated tissues. Due to the often heterogeneous nature of glioblastoma tissue, which may present with peritumoral edema and necrotizing areas, accurate soft tissue modeling is highly necessary for treatment planning and qualitative clinical analysis of coagulation progress.

**Figure 2:**
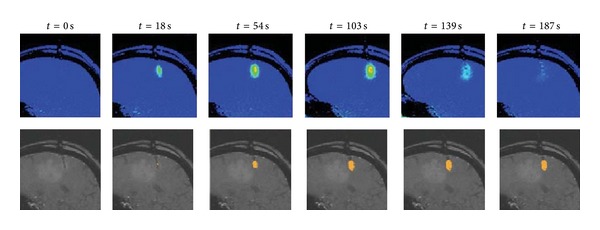
The commercial Visualase system allows the display of thermal distribution and an approximation of damage during the treatment [6]. Treatments including nanoparticle therapy would require much more complex modeling, as well as accurate estimations of nanoparticle concentration in the tissue.

Systems for integrating laser equipment and MRI visualization for MRgLITT are commercially available. Visualase Thermal Therapy has been used in several Phase I clinical trials treating tumors in the brain and in at least two studies where glioblastoma has been included. Working at wavelengths from 800 to 1064 nm, the system is capable of controlling laser ablation and simultaneously performing analysis of real-time MRTI information in order to output a false-color temperature map and a “damage map” estimated by the Arrhenius damage equation. The system allows for tunable control points to be set such that the laser deactivates if a certain temperature threshold is reached. See Figure 2.

#### 2.2.2. Clinical Results

In the majority of available clinical studies where LITT has been used to treat glioblastoma, most patients who were candidates for standard surgical, chemotherapeutic, and radiotherapeutic treatments had already undergone these procedures. Though initially labeled as alternative or salvage therapy to be attempted after all other therapies have been maximized, there have been some cases where LITT has been used as a primary debulking procedure on the basis of surgical inaccessibility, including at least one patient with glioblastoma [9].

The earliest clinical trial specifically addressing LITT treatment of glioblastoma was in 2006, where 16 patients, all presenting recurrent GBM and all nonsurgical candidates, were administered LITT using a 1064 nm Nd:YAG laser source coupled into a quartz fiber (length 12 m, ID = 400 *μ*m) [4]. The optical fiber used the diffusing tip to direct light emission into an ellipsoid profile. The fiber was positioned in the tumor using MR-guidance and a laser output of 6 W was selected. Thermal necrosis was induced in a ~2-3 cm diameter, with the axial length along the fiber being ~1 cm longer than the fiber itself. The thermally affected area extended ~3-4 cm in diameter around the necrotized tissue. Only one fiber was used in each treatment, though some patients received repeated irradiations. No patient required postoperative intensive care.

**Figure 3:**
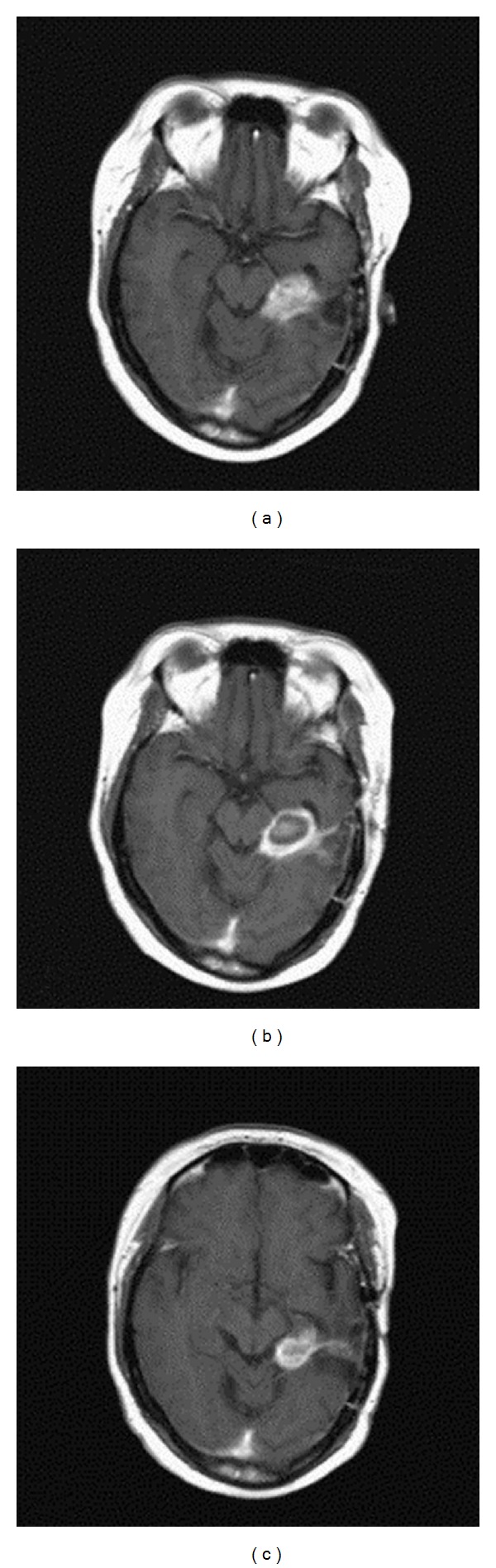
MR imaging of treatment progression of a recurrent GBM patient [4]: (a) Preoperative state and (b) 24 hours after LITT. A characteristic “ring” can be seen, indicating that necrosis has been achieved in the center of the tumor mass. (c) 17 months after LITT, tumor volume is reduced.

The mean tumor volume treated was 21.6 ± 18.6 cm^3^, with an average of 9.4 ± 3.3 kJ of energy delivered per tumor. Generally, tumors after irradiation exhibited a defined area of coagulation, which decreased in volume over time (e.g., see Figure 3). This is consistent with the results from a previous investigation by the same group [3]. The median overall survival time after diagnosis of recurrence was 9.4 ± 1.3 months, a substantial increase over the natural history survival prediction of <5 months. The median survival time after the first portion of the study (10 cases) was 6.9 ± 1.7 months; the second portion of the study demonstrated an increase in survival time to 11.2 ± 2.0 months. Reasons for this increase include self-reported selection bias in choice of patients with higher Karnofsky Performance Status (KPS) and smaller tumor size [4], though future studies would later qualitatively confirm the presence of a steep learning curve in the surgical procedure [8, 9].

Two recently published studies have used the Visualase system and very similar surgical methods in patients with glioblastoma. Both studies used a 980 nm diode laser with a cooling catheter (Visualase). They used real-time MRTI feedback in order to monitor the thermal ablation zone and used software to set specific boundary conditions for temperature on the ablation area.

**Figure 4:**
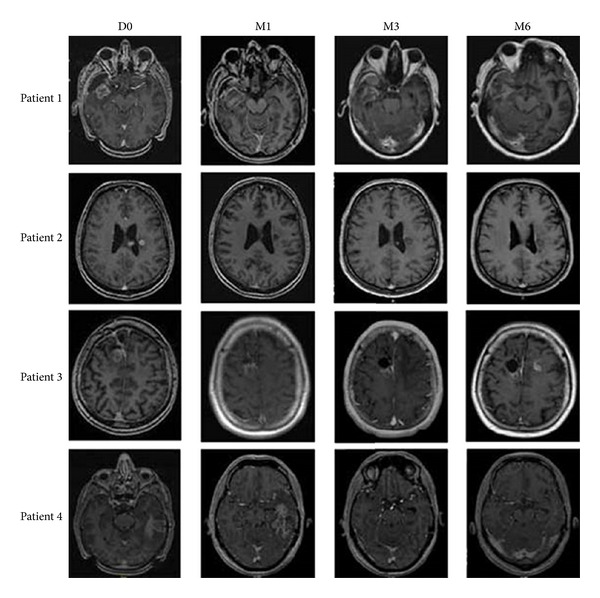
MR imaging of four patients with recurrent glioblastoma, following LITT on the day of treatment (D0), one month following (M1), three months following (3 M), and six months following (M6). Recurrence was observed in all patients, with a mean progression-free survival time of 37 days. Though LITT is suited for addressing many tissue types and tumor shapes/sizes, GBM masses are often diffuse in brain tissue. In order to discriminate between healthy and diseased tissue, highly specific therapies are desirable. Though more developed surgical methods may help in an immediate sense, reliance on simple hyperthermia outside the zones of ablative heat may not be sufficient for addressing microtumors seen in GBM.

The first study examined glioblastoma in four patients that had recurrent tumors after surgical resection. The fiber was positioned with MR-guidance and LITT was administered at 10–15 W for 30–180 seconds. The average tumor volume was much smaller than in the previous GBM recurrence study, with the largest tumor treated at ~5.5 cm^3^ [8]. The mean time of survival after LITT was 37 days. See Figure 4. A notable inference from this particular article notes the lack of correlation between the amount of tissue destroyed at the initial tumor site and the later recurrence of a tumor at that same site [8].

The second study examined 20 patients with highly variable tumors in various stages of treatment and progression [9]. LITT was administered at an average of 11.0 ± 1.4 W for an average of 13.9 ± 10.7 minutes. If the catheter could be inserted in the long axis direction of a tumor, multiple ablations along the same entrance channel could be performed. The mean tumor size treated was 7.0 ± 9.0 cm^3^. Mean survival time was not listed as the study was focused on the procedural aspects of the treatment. Investigators reflected on the presence of a steep learning curve in applying this treatment and recommended that this treatment be reserved for lesions less than 3 cm in diameter that are well circumscribed and noninfiltrating. However, the article does not provide a detailed list of tumor volumes and the investigators note that the size recommendation is based on experience and that most tumors treated were of a diameter less than 2 cm.

Recently, [19] a first-in-humans Phase I clinical trial by Sloan et al. used the NeuroBlate system for recurrent glioblastoma multiforme. The NeuroBlate system, previously known as the AutoLITT system, in conjunction with MRI thermometry, reports the temperature of tissue and thermal dose, providing feedback to the surgeon to tailor tissue coagulation. The NeuroBlate software determines the likelihood of cell death in the monitored region. Ten patients who had an average age of 55 and average KPS score of 80 were treated. Three levels of dose were administered. An external review board judged the need for dose escalation as warranted by toxicity level. A drop in KPS score measures toxicity level. The mean tumor volume was 6.8 ± 5 cm^3^. The median survival was 316 days. In conclusion, the NeuroBlate system represents new technology for controlled LITT in recurrent GBM.

Positron emission tomography (PET) has also been used recently to monitor the metabolic activity of the tumor before and after LITT treatments [10]. In a single case study, a 1064 nm laser was used to irradiate several points in a large glioblastoma in a 58-year-old patient. PET analysis determined that there was significantly less metabolic activity in the tumor area, in contrast to findings described by the contrasting area shown in the MRI. Future research may include PET scanning in order to better characterize the metabolic activity of the tumor before and after LITT treatment, as it may confirm assumptions derived from visual MR feedback and elucidate questions concerning the viability of local tissue after surgery [10].

## 3. Future Techniques Relevant to Glioblastoma

The investigation of LITT as a treatment for glioblastoma is inherently limited by the application of local methods to a diffuse disease. In many respects, LITT by itself cannot be considered much more than palliative treatment when regarded through the lens of invasive and widely distributed cancers. In contrast, the development of nanoscale technologies as a method of specific, cell-by-cell attack for cancerous tissue is highly significant to the future of cancer therapies of all types. Though targeting methods are still under investigation, glioblastoma is especially suited to nanoscale treatments as several reliable targeting methods have been developed for this disease *in vitro.* Of particular interest is the large subtype of GBM tumors which overexpress epidermal growth factor receptor (EGFR), providing a feasible target for many kinds of nanoparticle therapies [20]. Other targeting methods include monoclonal antibody tagging, which may be specific to a wide range of cell types, including GBM-specific CD133^+^ cancer stem-like cells [21].

Many preliminary studies have demonstrated that nanoparticles sensitive to irradiation in the NIR therapeutic range may be used for inducing hyperthermia in specific cells. LITT, then, may act as a method of delivering laser irradiation to nanoparticle-loaded tissues. In the context of use for cancer therapy, nanoparticles artificially increase the absorption in tissues, thus assisting the resultant heat and formation of necrosis when irradiated. In the case of targeted therapies, absorption is increased specifically in the targeted cell, thus preferentially heating that cell. This may be particularly useful for glioblastoma, which can develop tumor micromasses in a diffuse manner, including around arteries.

### 3.1. Preclinical Methods

Though not specifically focused on treatment of GBM, there have been several attempts in nanoparticle-assisted laser ablation in animal models, including an orthotropic canine model examining brain tumors, using gold shells surrounding silica cores or “gold nanoshells.” Gold nanoshells are highly relevant to laser therapeutics as their plasmon resonance may be tuned to a value in the NIR spectrum (in this case, 780–800 nm). Gold is also highly inert in the body, with no risk of oxidation or toxicity. In this study, 144–150 nm gold nanoshells coated with polyethylene glycol (PEG) when injected intravenously were shown to selectively accumulate in tumors due to the enhanced permeability and retention effect seen in tumor vasculature [22]; even without antibody conjugation or other targeting methods, this method has traditionally shown good selectivity and accumulation in tumors over healthy tissue. A 15 W gallium arsenide diode laser (*λ* = 810 ± 20 nm) paired with a cooling catheter (Visualase Cooled Laser Application System) was used due to the proximity to the absorbance peak of the gold nanoshells and the minimization of absorbance in oxyhemoglobin and deoxyhemoglobin [23]. Canine transmissible venereal tumor (cTVT) cells were injected into the brain to form the tumor model. The Visualase system was used to interface with the MRI and monitor thermal changes in the brain. The laser was used at 3.5 W for 3 minutes on average. Irradiation was repeated in healthy tissue on the opposite hemisphere of the brain. The nanoparticles assisted the thermal ablation to achieve ablative temperatures of 65.8°C, where the normal tissue only experienced heating up to the ineffective hyperthermic temperature of 48.6°C. After a healing period, the tumors were harvested and analyzed for nanoparticle content, demonstrating good distribution in the ablated tissue.

**Figure 5:**
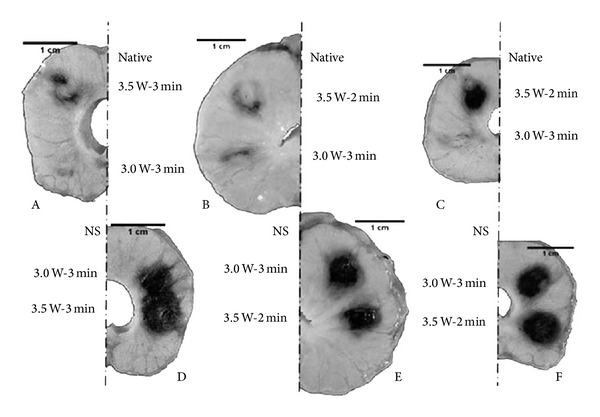
Dissection of formalin-fixed canine prostate tissue. Darkened areas indicate areas of thermal lesions induced at 3.0 or 3.5 W for 2 or 3 minutes. Sections A, B, and C indicate native tissue and sections D, E, and F indicate nanoparticle-laden tissue. 150 nm gold nanoshells were injected directly into the parenchyma of the prostate. It was found that lesion size grew with increasing power when applied to native tissue and that nanoshell application significantly increased the lesion size, but not with respect to power [23].

A further study by the same group was conducted in the prostate in a canine model, where a similar technique was used to demonstrate higher volume of necrosis at the same exposure and laser power in a nanoparticle-laden tissue compared to native tissue. The same laser applicator and cooling catheter (Visualase) were used for MRgLITT. Gold nanoshells were injected directly into the parenchyma of a single hemisphere of the prostate, using the bilateral tissue portion as a control. The applicator was passed through both hemispheres, with laser settings of 3–3.5 W for an irradiation time of 2-3 minutes. Cross-sections of the dissected prostrate (see Figure 5) demonstrated a much larger treatment area in the nanoparticle-laden tissue, with much more distinct and self-limited lesion boundaries [24].

This could indicate that some lasers, specifically those below 980 nm, may be better suited for nanomaterial excitation than for direct ablation itself. They may also be more suited for use in tissues with high perfusion due to their smaller absorptions of oxy- and deoxyhemoglobin. The exclusion of this particular fact in a choice of laser might be particularly inefficient for the brain, regardless of the speed of lesion development, considering that perfusion provides the largest challenge to heating efficiency in the brain, and thus high absorbance in blood tissue should be avoided.

Particles accumulate in the tumor by virtue of the enhanced permeability and retention effect. When tumors accumulate nanoparticles this way, they can be efficiently ablated with high selectivity over surrounding healthy tissues [23, 24]. In the case of the 150 nm gold nanoshells used in the canine brain tumor model, TEM imaging demonstrated that tumors accumulated along capillary walls. Though fenestrated vasculature is characteristic of many GBM tumors, these therapies alone would likely not provide the cell-by-cell specificity necessary for avoiding healthy cells in a diffuse mass. Furthermore, accumulation via the enhanced permeability and retention effect may provide less distributed nanoparticle mass across the tissue, giving uneven resultant heating effects. However, this type of nanoparticle-assisted therapy allows a less ablative dose to translate into an artificially higher-heat ablative therapy in a self-limited lesion size [24].

### 3.2. *In Vitro* Methods

Gold nanorods and other gold nanostructures continue to be investigated in many *in vitro *applications, including in GBM-specific cell lines [25]. Gold nanorods are often regarded as superior to nanoshells (which may exceed 150 nm in diameter [23]) with regard to size and cross-sectional profile in tissue. According to Mie theory, the ratio of scattering to absorption increases with size, so smaller nanoparticles are preferred in processes such as LITT where absorption is greatly preferred [25].

**Figure 6:**
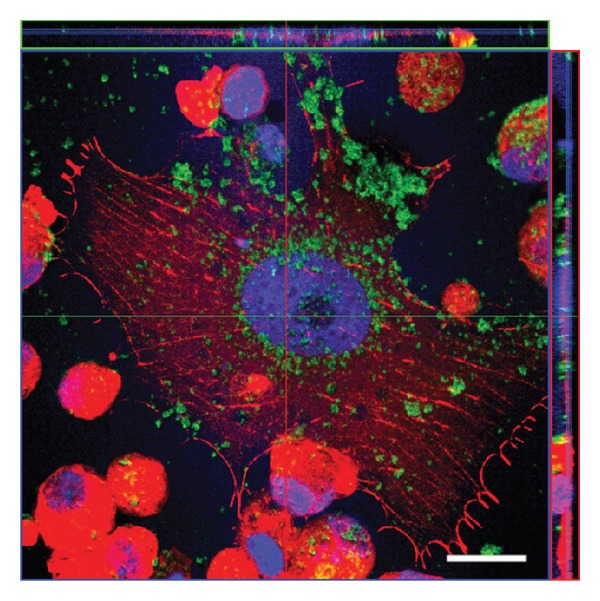
Confocal imaging of gold nanorod uptake in 1321N1 human brain astrocytoma cells after 12-hour exposure to 36 *μ*g/mL. Green coloring corresponds to the gold nanorods. The image effectively demonstrates that nanorods can be internalized into glioblastoma cells, including in the nucleus. Future investigation may elucidate the impact of internalized particles compared to nonspecific tissue-accumulated particles with regard to specificity in LITT treatments [24].

Gold nanorods were used to induce death in a glioblastoma cell line (1321N1 human brain astrocytoma). The cells were incubated with the gold nanorods at varying concentrations in order to achieve this. It was determined that gold nanorods were internalized by tumor cells by confocal microscopy (see Figure 6) and transmission electron microscopy (TEM) and that overall viability of the cells decreased with exposure to large concentrations of nanorods for periods longer than 20 minutes. After irradiation with an 808 nm source at 1.2 W for 20 minutes, significant cell death was seen in the nanoparticle-laden cells, but not in either of the irradiation-free or nanoparticle-free controls [25].

Though gold nanostructures are by far the most investigated particle type with regard to NIR irradiation in biological application, new NIR-sensitive substrates continue to be produced that may provide a more cost-effective alternative to gold nanoparticles. Potential nanoparticle alternatives include specialized carbon nanotubes [21], which have been used to specifically target CD133+ GBM cells. Other NIR-sensitive particles include reduced graphene oxide particles [26] and low band gap polymers [27].

## 4. Conclusions

Surgical debulking, by both operational resection and by LITT, does not yet provide a significant solution to mortality in GBM, see Table 1. However, LITT still proves to be a promising therapy for palliative care as it can significantly reduce the risk and discomfort caused by current resection surgeries—potentially providing a comparable extension of survival, but with a minimally invasive therapy that may be completed in a single day. LITT is also amenable to multiple treatments and to treatment of deep-seated and otherwise inaccessible tumors, which have limited alternate options for cytoreductive therapy.

In comparison to Gamma knife (GK) it is a more flexible technique, lending itself to treatment of the liver, lung, bone, and prostate as referenced earlier. The basic design of GK lends itself to treatment of the brain only and also requires the placement of a stereotactic head frame, which is painful. Deep-seated tumors can be treated by GK but not tumors on the periphery. Abnormal cells are not removed by the GK technique but are only damaged; therefore the physician must monitor the disease from weeks to months whereas LITT can be monitored in real time.

It is evident that nanotechnology will shape much of future cancer therapies. LITT may be of great use to these therapies if it can introduce laser irradiation into areas not otherwise accessible, such as those seen in deep-seated and diffuse GBM.

It is already evident that the type of laser used for this therapy may not necessarily be dependent on the amount of conductive heat that it may generate alone—immediate conclusions may be that longer ablation times with nanoparticle additions give superior results than shorter times which may produce less predictable thermal damage.

In these nanotherapeutic models, the value of the cooling catheter used in the laser applicator is particularly evident—the cooling of tissue medial to the laser applicator avoids the immediate coagulation usually seen at this point, resulting in a significant limitation in penetration depth. It can be postulated that further advances in cooling the laser applicator could be of great value to nanoparticle-enhanced ablation treatments, allowing much deeper optical penetration into the tumor. This could be uniquely useful to GBM in that deeper optical penetration could provide better access to diffuse cell structures.

## Figures and Tables

**Table 1:** Gives a summary of average values in the treatment of GBM.

Ref.	Laser wavelength (nm)	Number of patients	Tumor volume treated (cm^3^)	Energy delivered (kJ)	Survival time
[4]	1064	16	21.6 ± 18.6	9.4 ± 3.3	9.4 ± 1.3 months
[8]	980	4	5.5	0.3–2.7	37 days
[9]	980	20	7.0 ± 9.0	9.2	Not given
[19]	1064	10	6.8 ± 5.0	12.24 ± 9.7	316 days

## Abbreviations

GBM: Glioblastoma multiforme
LITT: Laser induced thermal therapy (also called laser induced interstitial thermotherapy, and variations thereof)
Nd:YAG: Neodymium-doped yttrium aluminum garnet
NIR: Near infrared
MRI: Magnetic resonance imaging
PET: Positron emission tomography
MRTI: Magnetic resonance temperature imaging
MRgLITT: Magnetic resonance-guided laser induced thermal therapy
KPS: Karnofsky Performance Status
cTVT: Canine transmissible venereal tumor.
